# Life history mediates mate limitation and population viability in self-incompatible plant species

**DOI:** 10.1002/ece3.963

**Published:** 2014-02-13

**Authors:** Peter H Thrall, Francisco Encinas-Viso, Susan E Hoebee, Andrew G Young

**Affiliations:** 1 CSIRO Plant IndustryGPO Box 1600, Canberra, Australian Capital Territory, 2601, Australia; 2 Department of Botany, La Trobe UniversityBundoora, Victoria, 3086, Australia

**Keywords:** Conservation, demographic rescue, gametophytic, inbreeding, mate availability, mating system, *S* alleles, simulation model, sporophytic

## Abstract

Genetically controlled self-incompatibility systems represent links between genetic diversity and plant demography with the potential to directly impact on population dynamics. We use an individual-based spatial simulation to investigate the demographic and genetic consequences of different self-incompatibility systems for plants that vary in reproductive capacity and lifespan. The results support the idea that, in the absence of inbreeding effects, populations of self-incompatible species will often be smaller and less viable than self-compatible species, particularly for shorter-lived organisms or where potential fecundity is low. At high ovule production and low mortality, self-incompatible and self-compatible species are demographically similar, thus self-incompatibility does not automatically lead to reduced mate availability or population viability. Overall, sporophytic codominant self-incompatibility was more limiting than gametophytic or sporophytic dominant systems, which generally behaved in a similar fashion. Under a narrow range of conditions, the sporophytic dominant system maintained marginally greater mate availability owing to the production of *S* locus homozygotes. While self-incompatibility reduces population size and persistence for a broad range of conditions, the actual number of *S* alleles, beyond that required for reproduction, is important for only a subset of life histories. For these situations, results suggest that addition of new *S* alleles may result in significant demographic rescue.

## Introduction

A central theme of evolutionary ecology is to understand how the interplay of genetic and demographic factors influences population dynamics, species persistence, and evolutionary processes. Quantifying the relative importance of such factors also has direct application in many areas. For example, management of small plant populations is a major concern in conservation biology with recent research efforts focusing on quantitative analysis of population viability. Major constraints considered to limit population performance in these situations fall into two broad classes. Demographic factors include asynchrony of flowering, pollinator limitation (Jennersten 1988; Oostermeijer et al. 2003), demographic and environmental stochasticity (Holsinger 2000), and Allee effects (Stephens and Sutherland 1999). Genetic factors that have been implicated include mutational meltdown (Lynch and Gabriel 1990; Lynch et al. 1995), inbreeding depression (Oostermeijer 2000; Frankham 2005), and hybridization (Rhymer and Simberloff 1996; Field et al. 2009).

It has been suggested that for those plant species with genetically controlled self-incompatibility (SI) systems (Richards 1997), reduced mate availability owing to loss of allelic richness (*S* alleles) at the incompatibility locus may also impose significant demographic constraints on small populations (Barrett and Kohn 1991; Byers and Meagher 1992; Frankham et al. 2002; Castric and Vekemans 2004), especially when acting in concert with ecological factors such as pollinator limitation (Busch and Schoen 2008; Young et al. 2012). Such direct genetically mediated demographic constraints may represent one of the few clear links between genetic diversity and population viability of immediate importance for plant population dynamics and long-term persistence. As such, self-incompatibility represents an example of a genetic trait whose phenotypic expression directly influences an ecological process (Hughes et al. 2008).

Genetically controlled, homomorphic, self-incompatibility occurs in approximately 50% of angiosperm families (Richards 1997; Igic et al. 2008). Allelic genealogy studies suggest that the two broad types of self-incompatibility systems, those exhibiting gametophytic control of mating phenotype, and those in which mating reactions are governed by the sporophytic generation, have evolved independently and possibly several times (Matton et al. 1994). Presumably, this convergent evolution reflects the advantages that such mechanisms provide in preventing inbreeding and its associated negative fitness effects.

Self-incompatibility systems function effectively in large populations where negative frequency-dependent selection acts to maintain large numbers of *S* alleles resulting in high mate availability. Emerson (1939) was the first to report the numbers of *S* alleles in a population, estimating that there were 45 different alleles in a population of *Oenothera organensis* (which exhibits gametophytic SI) consisting of 500 individuals. Since then, self-incompatibility loci have generally been shown to exhibit high within-population allelic richness (Richman and Kohn 1996; Lawrence 2000; Edh et al. 2009). In small populations, however, where founder events and genetic drift can erode allelic richness despite selection, self-incompatibility systems may limit mate availability and, through this, fertilization success and fecundity (Busch and Schoen 2008). This is more likely for sporophytic systems in which dominance relationships among alleles further reduce the effectiveness of frequency-dependent selection.

Several empirical studies have invoked low *S* allele richness as a possible cause of reduced seed set in small populations of self-incompatible plants: *Hymenoxys acaulis* var. *glabra* (DeMauro 1993), *Helenium virginicum* (Messmore and Knox 1997), *Cochlearia bavarica* (Fischer et al. 2003), *Ranunculus reptans* (Willi et al. 2005), and *Linnaea borealis* (Scobie and Wilcock 2009). Recently, Young and Pickup (2010) found positive relationships between population size, *S* allele richness, and mate availability in populations of the endangered daisy *Rutidosis leptorrhynchoides* that has a sporophytic self-incompatibility system. In this case, populations with few *S* alleles also exhibited elevated interplant variance in male and female fitness components, as expected under mate limitation (Byers and Meagher 1992), and reduced long-term viability as assessed by matrix projection models (Young et al. 2000).

Both analytical and computer simulation models have been used to examine how population size and gene flow interact to determine *S* allele diversity (Imrie et al. 1972; Schierup 1998; Castric and Vekemans 2004; Kirchner et al. 2006; Cartwright 2009; Levin et al. 2009) and how this affects mate availability and reproductive success in subdivided populations (Kirchner et al. 2006; Wagenius et al. 2007; Levin et al. 2009). Vekemans et al. (1998) used both deterministic and stochastic simulation approaches to show that gametophytic systems are less mate limiting than sporophytic systems and that dominance relationships among *S* alleles in sporophytic systems, and multiple pollination events strongly affect mate availability. Metapopulation models also suggest that effective among-population migration rates are likely to be higher for dominant than recessive alleles at SI loci (Schierup et al. 2000).

The combined results of these empirical and simulation investigations suggest that *S* allele number, frequency, and dominance relationships may have significant effects on the demography and genetic structure of plant populations through their influence on mating opportunities, and should be considered as important determinants of population viability for self-incompatible species. Some theoretical studies have begun to illustrate how demographic and genetic processes can interact to affect population viability (Kirchner et al. 2006; Wagenius et al. 2007; Levin et al. 2009). Here, we extend these analyses to explicitly investigate the influence of life-history and reproductive traits on individual fitness with the aim of elucidating broad predictive relationships that may drive population performance. For example, the relative impact of different mating systems is likely to vary with plant life-history features (e.g., birth and death rates) and this has so far not been considered.

Here, we develop a spatially explicit individual-based simulation approach which extends previous empirical and modeling studies to simultaneously examine genetic and demographic effects of both gametophytic and sporophytic SI on population viability. The important point of difference between these two SI systems is that in the gametophytic system, the pollen mating phenotype is controlled by the haploid pollen genotype, while in the sporophytic system, the pollen mating phenotype is determined by the diploid male genotype. Sporophytic SI therefore generally results in more limited mate availability although this may be alleviated by the effects of dominance relationships among *S* alleles. In addition, we assess how the relative impact of these systems is influenced by variation in major life-history parameters and the diversity of alleles at the SI locus. This integrated approach has several important advantages. First, we can identify sets of conditions where demographic or genetic factors are more limiting, which has practical implications for conservation management. Second, model predictions can be explicitly linked to empirically measureable population parameters (e.g., allelic richness, levels of correlated paternity, variance in seed set). Finally, it may be possible to identify suites of life-history traits that make species more demographically sensitive to changes in *S* allele richness.

## Computer Simulations

### Biological assumptions

To investigate population viability in relation to self-incompatibility and life history, the model allows plants to vary in reproductive capacity and lifespan (i.e., annual to long-lived perennials). Seed formation following a mating event is determined by a single SI locus with variable numbers of alleles. In the results presented here, this was varied from three (the minimum needed for there to be at least the possibility of successful mating events to occur) to 50, which approaches the maximum level of diversity seen at the population level in many species (Lawrence 2000; Castric and Vekemans 2004). The model also assumes five unlinked variable neutral loci, each with five alleles. In addition, the model assumes diploid genetics and that individuals can act both as pollen donors and as females.

The primary aim of this study was to gain understanding of how these major life-history features jointly influence variation in ecological factors associated with individual fitness (e.g., mean and variance in numbers of compatible mates and pollen donors, per capita seed production), as well as to examine how shifts in the mating system might interact with potential fecundity and death rate to impact on frequently measured genetic parameters (e.g., allelic richness at the SI locus and neutral loci, observed [*H*_0_] and expected [*H*_E_] heterozygosity, and the inbreeding coefficient [*F*_IS_]). We therefore use an individual-based approach to characterize variables that are frequently measured in field studies and investigate how these relate to population viability and persistence. The model assumes no fitness consequences of selfing (i.e., when using a self-compatible mating model as a baseline comparison), as the goal is not to examine the conditions under which self-incompatibility evolves, but rather to focus on the implications of SI for plant population viability.

### General model structure and initial conditions

The simulations (written in C++ using the GNU Scientific Library) consist of a two-dimensional (100 × 100) grid, with absorbing boundaries, as this scenario best mimics natural populations of finite size, beyond which propagules are essentially lost from the system. The fraction of sites suitable for occupancy can be varied – these are assigned randomly at the beginning of each run (this value was fixed at 0.25 for the results reported here). At the start of each run, genotypes were randomly assigned to each of 200 individuals (with the constraint that individuals must be heterozygous at the SI locus). These were randomly placed in a subset of the available spatial locations. In addition to spatial location and genotype, the simulation also tracks individual ages.

In each time interval, for each reproductive adult (the minimum age of reproduction was fixed at 1, thus all individuals were considered to be adults), the total number of possible pollen donors (including both compatible and incompatible) within pollination distance is determined. The spatial scales of both pollen and seed movement can be varied in the simulation, however, for the purposes of this study, these were fixed at 10 and 5, respectively (e.g., for each adult, potential pollen donors were chosen from the pool of individuals within 10 units or less).

To calculate per capita ovule production, we assumed a linear relationship with individual age (*A*), such that *M* = the age of first reproduction, *L* = the expected lifespan (1/*d*, where *d* = death rate), and *b*_0_ = maximum per capita ovule production. The number of ovules produced by an individual (*Ov*) is thus (*b*_0_*d*) *A* when *A* is less than or equal to *L*, otherwise *Ov* = *b*_0_ (this is reasonable for most plants, particularly long-lived perennials, where reproductive output does not increase indefinitely with size). To ensure that the number of ovules produced is at least 1 at the minimum reproductive age, *b*_0_ was constrained to be greater than 1/*dM*. For each ovule produced by an individual, a random pollen donor was chosen from the pool of possible mates (haploid ovule and pollen genotypes were randomly constructed from the diploid parents), and then, compatibility was determined based on whether the mating system was self-compatible (i.e., full compatibility across all individuals), gametophytic SI, sporophytic codominant, or sporophytic dominant (dominance was assumed to be paternally controlled as this is the most commonly seen form; Richards 1997). In the case that the ovule and the chosen mate are incompatible, then the ovule is aborted and another ovule is chosen for mating. For each plant, seeds generated from successful fertilizations were then dispersed randomly within the seed dispersal range. This approach simulates pollination by multiple pollen donors at the maternal level, which has been shown to be important in generating biologically realistic fertilization probabilities (Vekemans et al. 1998). Seeds dispersing outside of the grid or to cells already occupied by adults were assumed to have been lost. Once all individuals had mated and reproduced, and seeds dispersed to empty cells, adult death occurred with a fixed probability. Following this, in each cell where one or more seeds had landed, a single individual was randomly chosen to germinate.

### Simulation experiments

To explore the demographic and genetic consequences of variation in self-incompatibility system, *S* allele richness and life history, the following parameters were varied systematically in the simulation model: mating system (as noted above), the number of *S* alleles (3, 5, 10, 25, 50), death rate (0.05, 0.1, 0.2, 0.3, 0.5, 1.0), and per capita ovule production rate (10, 25, 50, 75, 100). Note that even very high ovule production values translated into realistic numbers for per capita production of surviving offspring. The situations we investigated simulated life histories ranging from biennials to long-lived perennials and covered a wide range of reproductive values.

Each simulation ran for a maximum of 500 generations – for runs in which the population persisted for the full duration, both demographic and genetic patterns generally stabilized within the first 100–200 generations. For each set of the parameter combinations outlined above, 100 random runs were conducted, and results averaged across runs for the following demographic variables at each generation: total number of reproductive plants, proportion of reproductive individuals within pollen-flow range that were of compatible genotypes at the SI locus (mate availability), number of seeds/plant and its variance. Mate availability was calculated as the average number of compatible individuals. Similarly, information was gathered at each generation for several genetic variables: number of alleles per neutral locus (across loci and individuals), number of alleles at the SI locus, observed and expected heterozygosity, and the fixation index (*F*_IS_). In addition, for each set of parameter combinations, the percentage of runs that persisted for the full 500 generations was recorded, as well as average population persistence time (number of generations).

## Results

### Population viability

**Figure 1:**
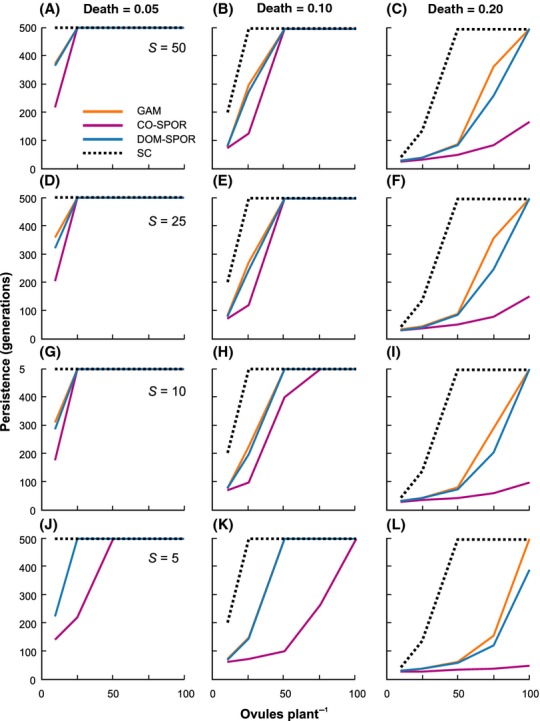
Population persistence under different breeding systems as a function of death rate, ovule production rate, and number of alleles at the self-incompatibility (SI) locus. Moving from left to right for the three columns of graphs, death rate was fixed at *d* = 0.05, 0.10, and 0.20, respectively. Moving vertically down each column, the number of *S* alleles was fixed at 50, 25, 10, and 5, respectively. For each graph, ovule production rate was varied (*b*_o_ = 10, 25, 50, 70, 100). Each graph shows persistence for the gametophytic (solid orange lines), sporophytic dominant (solid blue lines), and sporophytic codominant (solid purple lines) SI systems. Note that gametophytic and sporophytic dominant SI systems show very similar persistence for *d* = 0.05 and 0.1, and in these cases, the lines overlap. Results for the self-compatible case are plotted on all graphs for comparison (dashed lines).

**Figure 2:**
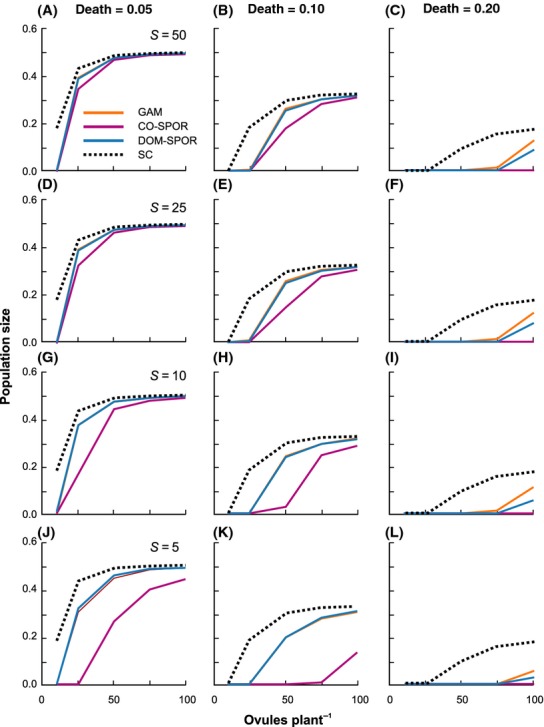
Population size after 500 generations under different breeding systems as a function of death rate, ovule production rate, and number of alleles at the self-incompatibility (SI) locus. Moving from left to right for the three columns of graphs, death rate was fixed at *d* = 0.05, 0.10, and 0.20, respectively. Moving vertically down each column, the number of *S* alleles was fixed at 50, 25, 10, and 5, respectively. For each graph, ovule production rate was varied (*b*_o_ = 10, 25, 50, 70, 100). Each graph shows population size for the gametophytic (solid orange lines), sporophytic dominant (solid blue lines), and sporophytic codominant (solid purple lines) SI systems. Results for the self-compatible case are plotted on all graphs for comparison (dashed lines). Note that gametophytic and sporophytic dominant SI systems show very similar population sizes for *d* = 0.05 and 0.1, and in these cases, the lines overlap. Population size is expressed as the proportion of suitable sites occupied.

Negative effects on population persistence were generally more dramatic with increasing death rates and lower birth rates. Regardless of which self-incompatibility system was assumed, or the level of allelic richness at the SI locus, when death rates were 0.3 or greater, populations uniformly went extinct within a few generations (<50). For lower death rates (*d* ≤ 0.2), there was significant variation among SI systems with respect to persistence and population size, and clear effects of genetic diversity at the SI locus (Figs. 1,2). Regardless of death rate, ovule production or *S* allele richness, the sporophytic codominant system always resulted in the lowest population persistence and size. The gametophytic and sporophytic dominant systems generally behaved in a similar fashion, with persistence times and population sizes intermediate to the sporophytic codominant and self-compatible situations.

### Time to extinction

For very low death rates (*d* = 0.05), population persistence was only significantly reduced relative to the self-compatible case when ovule production was also very low (*b*_o_ < 25). *S* allele richness had no effect on population persistence for either the gametophytic or sporophytic dominant systems. However, for the sporophytic codominant system, populations initiated with only five *S* alleles had an increased probability of going extinct for substantially higher rates of ovule production (*b*_o_ < 50; Fig. 1J).

For somewhat higher death rates (*d* = 0.1–0.2), limitations on population persistence imposed by self-incompatibility were manifested over a broader range of ovule production rates and levels of *S* allele richness. At the lowest level of ovule production (*b*_o_ = 10), these death rates also led to reduced population persistence time for the self-compatible case. Interactions between *S* richness, ovule production, and mating system also became apparent. These were most obvious for the sporophytic codominant system where the effect of reduced *S* allele richness was strongly dependent on ovule production. For example, when *d* = 0.1 and the ovule production rate was 50, reducing *S* allele richness from 25 to 5 decreased persistence time by 80% (i.e., from 500 to 100 generations; compare Fig. 1E and K). For the same death rate, but with 75 ovules produced per plant, then the effect of reducing *S* allele richness was only a 50% drop in persistence, and no reduction was observed when the ovule production rate was 100. This interaction between ovule production and *S* allele richness was evident to a lesser extent for the gametophytic and sporophytic dominant systems.

Further increases in death rate (*d* = 0.2) resulted in a general and dramatic reduction in population persistence for all self-incompatibility systems, with long-term population viability only being achieved for the highest rates of ovule production and then only for the gametophytic and sporophytic dominant systems. These two SI systems were also strongly influenced by *S* allele richness but, in contrast to the case when *d* = 0.1, effects were most evident at higher ovule production rates (*b*_o_ = 50–75; Fig. 1). This was not the case for the sporophytic codominant system, where persistence was uniformly low, and extinction rates were little affected by either *S* allele richness or ovule production.

### Population size

Although, in the simulation results presented here, a fixed 2500 of the 10,000 sites represented suitable habitat, in no case did the percentage of sites occupied exceed 50% of the available habitat. This indicates that constraints on population growth were demographic and genetic, rather than being due to the simulation structure itself (i.e., there were always available sites for plants to colonize). Trends in population size after 500 generations paralleled the patterns seen with respect to persistence, although the effects of decreases in ovule production were more pronounced, even for the self-compatible case (Fig. 2).

Irrespective of death rate, for the self-compatible case, there was a clear asymptotic relationship between ovule production and population size, such that beyond a certain level, further increases in ovule production did not result in a higher percentage of the available sites being occupied by adults. Moreover, this asymptote itself decreased with increasing death rates. For low death rates (*d* = 0.05, 0.1), this pattern was also evident for the three SI mating systems, but significant reductions in population size relative to the self-compatible case were observed when *S* allele richness was 10 or less and ovule production lower than 75, particularly for the codominant sporophytic case. As with persistence time, the interacting effects of *S* allele richness and ovule production were most evident when *d* = 0.1. When *d* = 0.2, the magnitude of reductions in population size relative to the self-compatible case was great enough that the asymptote would only be reached for ovule production rates greater than 100, regardless of which SI mating system was assumed.

Taken together, these results indicate that at very low death rates (*d* = 0.05), population sizes are generally large enough that neither demographic nor genetic factors impact significantly on persistence, except when both ovule production rates and *S* allele richness are very low. In contrast, at death rates of 0.2 or greater, demographic limitations reduce population size to the extent that even with high ovule production rates, large numbers of *S* alleles are necessary for long-term persistence. However, even in this case, population sizes remain small. It is only at intermediate death rates (*d* = 0.1) where population sizes and persistence can be strongly influenced by both demographic and genetic factors.

### Demographic and genetic dynamics

Changes in persistence and equilibrium population size are the most visible consequences of the impacts of shifts in major life-history parameters (e.g., birth and death rates) on a range of underlying demographic (mate availability, seed set, male/female fitness) and genetic (loss of *S* alleles, *H*_0_, *F*_IS_) factors. In self-incompatible mating systems, these variables can interact to severely reduce population sizes, thereby resulting in decreased long-term persistence through stochastic extinction events. The results presented above indicate that demographic and genetic effects are both important for intermediate death rates and low to intermediate levels of fecundity and *S* allele richness. To examine in detail how these interact to impact on viability, in this section, we examine population dynamics for situations where *d* = 0.1, *b*_o_ < 75, and the number of *S* alleles <50. We focus on comparisons between the gametophytic and sporophytic codominant situations as these bracket the extremes in terms of how restrictive SI systems can be, because, with few exceptions (see Gametophytic vs. Sporophytic Dominant Self-Incompatibility below), the sporophytic dominant and gametophytic cases were generally similar.

### Mate availability and *F*_IS_

**Figure 3:**
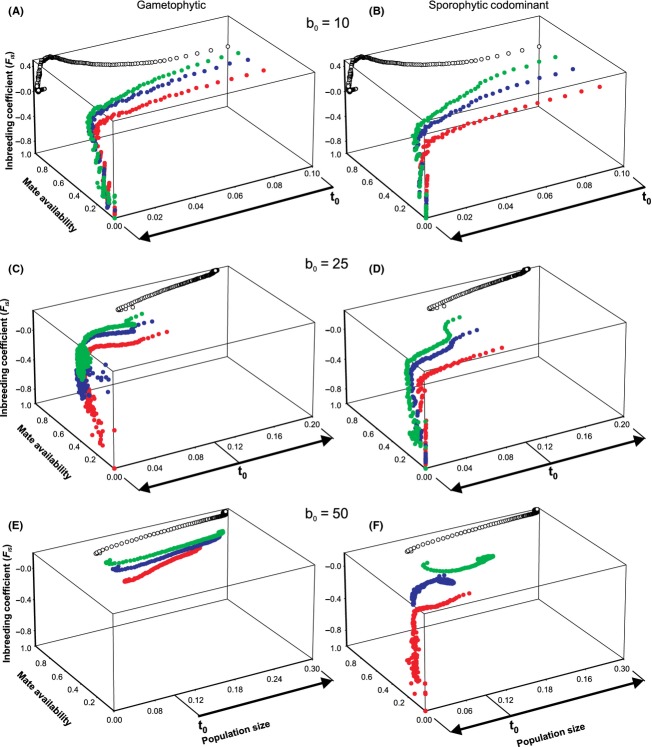
Dynamical relationship between population size, mate availability, and the inbreeding coefficient (*F*_IS_) for different levels of *S* allele richness. The left-hand column of graphs shows results for the gametophytic self-incompatibility system, and the right-hand column of graphs shows results for the sporophytic codominant system. Ovule production rates were fixed at 10, 25, and 50 for the top, middle, and bottom rows of graphs, respectively. Changes in population size equate directly with dynamics through time, with arrows indicating the temporal direction of changes from the starting point (*t*_0_). Trajectories represented by filled green circles are for *S* = 50, while filled blue and red circles represent dynamics for *S* = 25 and *S* = 10, respectively. Open black circles show dynamics under the self-compatible scenario. For all results shown, the death rate (*d*) was fixed at 0.1.

When ovule production was very low (*b*_o_ = 10), *S* alleles were rapidly lost from populations regardless of breeding system or initial *S* allele richness. In these situations, this reduction resulted in a severe restriction in local mate availability relative to the self-compatible case, very negative inbreeding coefficients (*F*_IS_) indicating strong disassortative mating (Fig. 3A and B), and extinction always occurred within 150 generations (Fig. 1).

Relatively small increases in ovule production (*b*_o_ = 25) were sufficient to ensure population growth and long-term viability of self-compatible populations but not for either the gametophytic and sporophytic codominant SI systems, for which population sizes still declined substantially from initial values (Fig. 3C and D). However, the influence of *S* allele richness on mate availability and *F*_IS_ was now dependent on breeding system. For the gametophytic system, increasing *S* allele diversity led to reduced disassortative mating and greater retention of mate availability (Fig. 3C). In contrast, the sporophytic codominant system continued to be severely mate-limited, regardless of the number of *S* alleles with which the simulation was initiated. Further increases in ovule production (*b*_o_ = 50) resulted in positive population growth for the gametophytic system regardless of *S* allele richness – mate availability never declined and *F*_IS_ was always either zero or slightly positive. However, *S* allele richness had dramatic effects on these parameters for outcomes in the sporophytic codominant case (Fig. 3F), ranging from extreme mate limitation and extinction (*S* = 10) to random mating and demographic increase similar to the self-compatible situation (*S* = 25).

### Seed set and variance in fitness

Changes in mate availability associated with population size jointly depend on breeding system and *S* allele diversity. In turn, the former exert their influence on population growth and persistence primarily through their effects on individual reproductive performance. This is most easily observed by examining the mean and variance of seed set.

**Figure 4:**
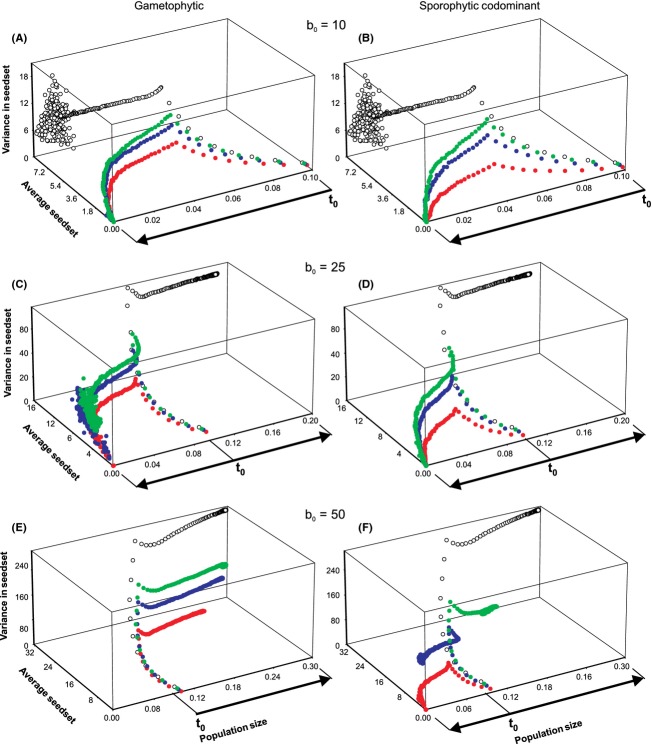
Dynamical relationship between population size, average seed set, and variance in seed set for females for different levels of *S* allele richness. The left-hand column of graphs shows results for the gametophytic self-incompatibility system, and the right-hand column of graphs shows results for the sporophytic codominant system. Ovule production rates were fixed at 10, 25, and 50 for the top, middle, and bottom rows of graphs, respectively. Changes in population size equate directly with dynamics through time, with arrows indicating the temporal direction of changes from the starting point (*t*_0_). Trajectories represented by filled green circles are for *S* = 50, while filled blue and red circles represent dynamics for *S* = 25 and *S* = 10, respectively. Open black circles show dynamics under the self-compatible scenario. For all results shown, the death rate (*d*) was fixed at 0.1.

In situations where low numbers of *S* alleles or low ovule production rates led to severe mate limitation (e.g., Fig. 4A, B, and D), then decline in population size was correlated with an initial increase in both the mean and variance of seed set. This phase of the dynamics was followed by a rapid decline in both variables as disassortative mating reduced mate availability to very low levels. This partly reflects the early development of an age-structured population (ovule production rates were assumed to be a positive function of plant age/size) and subsequently the buildup of fine-scale spatial genetic structure. Such structure initially provides increased opportunities for mating as local plant density increases, but as *S* alleles are lost and relatedness increases, the local pool of genetically compatible pollen donors ultimately becomes restricted.

The point at which the positive effect of increasing *S* alleles on mate availability is sufficient to maintain seed set and prevent extinction (Fig. 4C, E, and F) depends on breeding system and the maximum rate of ovule production. Higher rates of ovule production and gametophytic systems require fewer *S* alleles for population persistence than lower ovule production rates and sporophytic codominant systems. Just above these thresholds (Fig. 4C), the maintenance of mate availability in small populations is not reflected in a qualitative difference in the general nature of the dynamical relationship between population size and seed set. Instead, persistence of small populations when *S* = 25 is due to a continued low level of seed set. As ovule production increases further (and demographic constraints become less limiting), both qualitative and quantitative effects of *S* allele diversity on the relationship between population size and mean and variance in fecundity become apparent (Fig. 4F). In this case, *S* = 25 populations grow, while *S* = 10 populations persist at reduced size and *S* = 5 populations consistently go extinct.

### Gametophytic versus sporophytic dominant self-incompatibility

**Figure 5:**
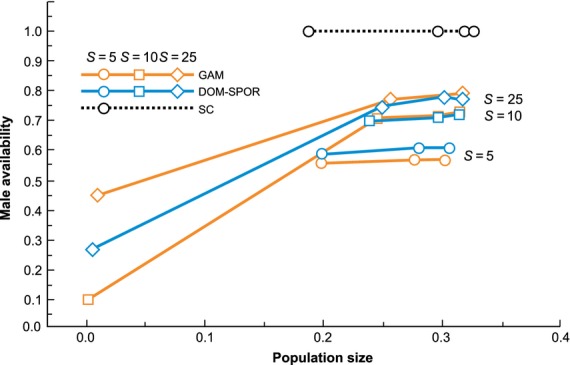
Comparison of relationship between population size and mate availability for populations with gametophytic (solid orange line) and sporophytic (solid blue line) dominant self-incompatibility under different levels of *S* allele richness: *S* = 5 (open circles); *S* = 10 (open squares); *S* = 25 (diamonds). Results show that when the number of *S* alleles is ten or greater, gametophytic systems have higher mate availability, but when there are fewer than five *S* alleles, sporophytic incompatibility is less limiting. This is probably owing to the generation of *S* locus homozygotes in the sporophytic system that cannot arise under gametophytic control. The self-compatible situation is shown with open black circles and dashed lines.

Previous theoretical studies (Vekemans et al. 1998) have suggested that sporophytic self-incompatibility systems should generally be more limiting in terms of mate availability than gametophytic systems due to the increased likelihood of matching *S* alleles between mates in sporophytic systems where the pollen mating phenotype is determined by the diploid male SI genotype. Our results indicate that under most conditions, these systems perform in a very similar fashion (although if there were differences, it was usually the sporophytic dominant system that was more limiting). However, at very low levels of *S* allele diversity (where population sizes are also small), our results demonstrated that mate availability was actually higher for the sporophytic dominant system than for the gametophytic system (Fig. 5). The most likely explanation for this is that dominance relationships among alleles in the sporophytic system permit the buildup of SI locus homozygotes. This is not possible with either gametophytic or sporophytic codominant self-incompatibility systems.

## Discussion

### Effects of self-incompatibility on population viability

Theoretical and simulation studies have suggested that self-incompatible breeding systems have significant potential to negatively impact long-term population fecundity and viability, particularly during population crashes and subsequent bottlenecks, or in the colonization phase of population growth (e.g., Byers and Meagher 1992; Vekemans et al. 1998; Wagenius et al. 2007; Hoebee et al. 2008). In these situations, impacts are likely to be primarily through severe reductions in mate availability and fertilization success owing to reduced numbers of *S* alleles, as has been demonstrated in a number of single species case studies (Fischer et al. 2003; Willi et al. 2005; Glémin et al. 2008; Scobie and Wilcock 2009; Leducq et al. 2010; Young and Pickup 2010).

Results from deterministic computer simulations of genetic dynamics indicate that in small populations, decreases in *S* allele diversity lead to reductions in average mate availability but an increase in the variance of available mates (Byers and Meagher 1992; Vekemans et al. 1998). Because these studies were focused explicitly on genetic rather than demographic processes, it is difficult to know whether these effects are likely to be significant relative to other population-level processes in natural systems. However, recent studies suggest that both demographic and genetic constraints operate in subdivided populations (Kirchner et al. 2006; Wagenius et al. 2007; Levin et al. 2009). The model we present explicitly integrates numerical and genetic dynamics in a biologically realistic spatial context, allowing us to directly evaluate the potential for self-incompatibility and life-history features to jointly impact on population viability relative to other demographic processes. Our results generally support the idea that, in the absence of inbreeding effects, populations of self-incompatible species will often be smaller and less viable than self-compatible species, particularly for shorter-lived organisms or where birth rates are low, although it must be remembered that the lack of inbreeding depression in the self compatible (SC) case may inflate this difference. Thus, one major conclusion is that longer-lived organisms such as trees can withstand negative genetic effects on reproduction better than short-lived species. However, for some ovule production and death rates, SI systems performed in a similar manner demographically to the self-compatible system, and thus, self-incompatibility does not automatically lead to reductions in mate availability.

It has been hypothesized that sporophytic self-incompatible systems should be more mate-limited than gametophytic systems (e.g., Vekemans et al. 1998; Castric and Vekemans 2004), and some simulation studies have shown this (Levin et al. 2009). As expected, our results showed that, under most demographically limiting conditions, the sporophytic codominant system performed worse than either the sporophytic dominant or gametophytic systems – in the former case, more severe restrictions on mate availability resulted in smaller populations that persisted for shorter periods of time (and in fact the available data indicate that fully codominant systems are rare in nature; Richards 1997). However, there were relatively few differences between the sporophytic dominant and gametophytic systems, although surprisingly under a limited set of conditions (e.g., when *S* allele richness was very low, *S* < 5), the sporophytic dominant system maintained marginally greater mate availability due to the production of SI locus homozygotes, leading to small increases in population size and viability over the gametophytic system. Again in this comparison, the lack of inbreeding depression in the model may inflate the observed difference as it means that there is no fitness cost associated with highly homozygous (inbred) individuals while they do gain a mating advantage.

### *S* allele richness and population viability

Given that under some conditions, self-incompatibility can have significant viability consequences for plant populations, it is important to ask when *S* allele richness (and the erosion of genetic variation at the SI locus) is of concern. This issue has been raised previously by several authors (Les et al. 1991; Young et al. 2000; Frankham et al. 2002). While possessing some form of self-incompatibility clearly reduces population size and persistence for a broad range of conditions, the actual number of *S* alleles is important for a more limited set of life histories. For example, when death rates are high (and populations are severely demographically limited), then adding new *S* alleles has little or no effect on viability. This is also the case when demographic constraints are absent (low death rates and intermediate to high birth rates – for these parameter ranges, no genetic limitations on population size were apparent). It is only for intermediate situations, where differences between SI systems also become more apparent, that *S* allele richness further impacts on viability (and even then, this is generally only seen when the number of alleles is relatively low).

**Figure 6:**
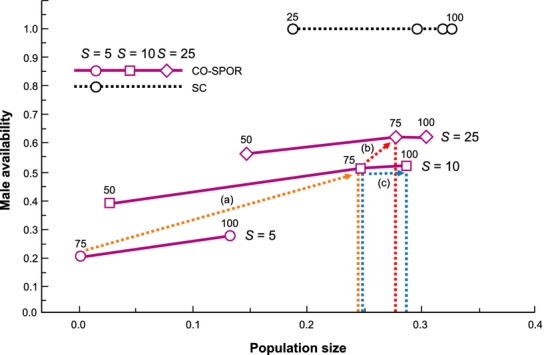
The influence of increasing the number of *S* alleles on population size in a species with sporophytic codominant self-incompatibility. When ovule production is low, increasing the number of *S* alleles from 5 to 10 has a strong positive influence on population size, indicating that genetic rescue is an effective management strategy (trajectory a – orange dashed arrow). Beyond this, however, increasing numbers of *S* alleles has much less effect (trajectory b – red dashed arrow), suggesting that demographic management will be more useful to promote population viability (trajectory c – blue dashed arrow). In this case, an effective increase in ovule production to *b*_o_ = 100, achieved in practice through the promotion of pollination or germination, is approximately 25% more effective in promoting population recovery.

The available empirical evidence indicates that most populations of SI species maintain quite high numbers of *S* alleles (Richards 1997; Lawrence 2000; Castric and Vekemans 2004). Nevertheless, our results also indicate that under at least some conditions, increasing population viability through addition of new *S* alleles may be the most effective approach to population management (Fig. 6) and there is some empirical support for this strategy. An extreme example is the threatened daisy, *Hymenoxys acaulis* var. *glabra*, which was shown to lack cross-compatible mating types in remaining populations; the recovery program explicitly focused on establishing new populations with a diversity of *S* allele types (DeMauro 1993). The potential for genetic rescue to increase fecundity through the addition of new *S* alleles has also been demonstrated experimentally for several other short-lived perennial herbs (*Ranunculus reptans*, Willi and Fischer 2005; *Rutidosis leptorrhynchoides*, Pickup and Young 2008; *Brassica insularis,* Glémin et al. 2008).

The effectiveness of such genetic rescue strategies partly depends on what type of self-incompatibility is present in a particular species. Based on theoretical considerations, it has been suggested that the efficiency of frequency-dependent processes in maintaining rare *S* alleles is likely to vary for the different SI systems (Vekemans et al. 1998; Levin et al. 2009). Our results (data not presented) showed that alleles were lost consistently slower in the gametophytic system than in either of the sporophytic systems (although overall the differences between sporophytic dominant and gametophytic SI were relatively small). The rate of loss (and the endpoint) also was dependent on the interaction between population size, mate availability, and seed set.

### Interactions between self-incompatibility, demography, and genetics

Overall, our simulation results showed that, dynamically, population declines (as a consequence of high mortality and/or low ovule production rates) are accompanied by a cascade of demographic and genetic effects. Some variables respond only very slowly to decreases in population size (e.g., *F*_IS_) with little apparent change over large size ranges. Generally, *F*_IS_ values were highest for intermediate sized populations, decreasing slightly for larger populations (but always positive in these cases). As population sizes became very small *F*_IS_ values often oscillated dramatically (sometimes between negative and positive values) before population extinction occurred, however, the general trend was toward negative values reflecting strong mate limitation and disassortative mating. The generation of significant negative values at the neutral loci as a result of disassortative mating suggests that physical linkage is not necessary for the *S* locus to exert considerable influence on the genotypic makeup of individuals. This extends previous results showing that loci linked to the *S* locus are not only less amenable to purging of deleterious alleles by selection (Glémin et al. 2001), but are also protected from the effects of random drift (Schierup et al. 2000; Ruggiero et al. 2008). Note that under gametophytic self-incompatibility, populations could become much smaller before going extinct than with sporophytic self-incompatibility, particularly with respect to the codominant case. In the latter, mate availability became limiting far sooner.

For other parameters (e.g., reduction of mate availability, variance in seed set), responses to changes in population size were much more predictable (i.e., we did not observe oscillations as for *F*_IS_). Interestingly, both average mate availability and variance in seed set were lowest for very small or very large populations. When populations are large, then mate availability is not limiting and all individuals can set seed, while the extreme limitation at small sizes results in generally low fecundity. Initial increases in seed set, even though population sizes are declining (Fig. 4), are primarily due to increases in local plant density through recruitment into empty sites and the development of age structure. However, as spatial genetic structure builds up further, local clusters of individuals become increasingly related (data not shown), particularly as *S* alleles are lost, and mate availability decreases again. Thus, not only are life-history characteristics important, but pollination and dispersal parameters are critical because of their impact on the buildup of local spatial genetic structure. Wells and Young (2002) present supporting evidence for this effect in the self-incompatible daisy, *Rutidosis leptorrhynchoides*.

### Results from empirical studies

Several empirical studies have examined aspects of the population biology of self-incompatible species, most commonly limits placed upon reproductive performance by low genetic mate availability. For example, Les et al. (1991) investigated the reproductive biology and genetic structure of a rare daisy, *Aster furcatus,* which has sporophytic self-incompatibility and primarily persists in small populations. Their results showed that the inbreeding coefficient (*F*_IS_) was generally negative in these situations, indicating an excess of heterozygotes. Our simulation results suggest that this observation likely reflects strong disassortative mating under severe mate limitation when *S* allele numbers are low. Based on this result, Les et al. (1991) suggested that severe bottlenecks (e.g., through habitat fragmentation) leading to the loss of incompatibility types may be a major threat to long-term persistence of species such as *A. furcatus*. Similar negative *F*_IS_ values have also been reported for several highly outcrossed tree species (Hall et al. 1994; Dayanandan et al. 1999; Degen et al. 1999; Lexer et al. 2000), indicating that this may be a common outcome for small populations of SI plants.

Detailed quantitative analyses of mating structure, demography, and genetic variation have been carried out in multiple populations of several rare plant species with different life histories: a woody shrub (*Grevillea iaspicula* (Hoebee and Young 2001)) exhibiting gametophytic self-incompatibility and two herbaceous perennials showing sporophytic dominant self-incompatibility (*Rutidosis leptorrhynchoides* (Young et al. 1999, 2000); *Arnica montana* (Luijten et al. 2000)). Results from these studies provide strong support for the dynamical patterns seen in our model. For example, in both *Arnica* and *Rutidosis*, negative *F*_IS_ values were consistently observed in populations of <50 plants (Young et al. 1999), which is within the size range at which our model predicts this should occur. Young et al. (1999) suggested that negative inbreeding coefficients might be due to an absence of homozygotes for low-frequency alleles. However, our analyses indicate that these patterns are much more likely to be due to severe mate limitation caused by the loss of *S* alleles. Further support for this interpretation comes from the observed high levels of correlated paternity and the strong differentiation between pollen and adult allele pools in small populations of *Rutidosis* (Young and Brown 1999; Young and Pickup 2010). Interestingly, the data presented in Young et al. (1999) also suggest that decreases in population size may have very little impact on *F*_IS_ until a threshold is reached, below which values decline very sharply. This pattern closely matches dynamical predictions for *F*_IS_ derived from our model (see Fig. 3).

*Grevillea iaspicula* is one of the few other self-incompatible species whose demography, mating system, and genetic structure have been intensively studied. This species persists in a few small remnant sites with population sizes generally less than 50 flowering plants. Simulation studies using the model described in this study again indicate close agreement with empirical observations of demographic and genetic population parameters. Predicted equilibrium population sizes ranged from 30 to 50 reproductive individuals (average number of reproductive plants within *G. iaspicula* populations = 29.6). The model also predicted negative inbreeding coefficients (ranging from −0.02 to −0.07), and observed values, based on allozyme data, ranged from −0.01 to −0.12 (Hoebee et al. 2008).

### Implications for population management

What are the practical implications for population recovery programs when the species concerned has a self-incompatible mating system? Several authors have demonstrated, using interpopulation crosses, a direct link between *S* allele diversity and mate availability which means there is real potential for demographic recovery through the addition of alleles to genetically depauperate populations (DeMauro 1993; Willi and Fischer 2005; Pickup and Young 2008). However, there has been little attempt to rigorously quantify how much of a fecundity increase is possible for a given number of *S* alleles, nor has the trade-off with demographic approaches (e.g., pollen augmentation, promotion of germination, elimination of competition) to increasing recruitment and survival been examined. These points are illustrated in Figure 6, which shows that when *S* allele diversity is very low, adding even a few new cross-compatible mating types can significantly increase equilibrium population sizes. However, once the number of *S* alleles is above 10–15, purely demographic strategies might be better, although the precise point at which one or the other approach is likely to be most effective will depend on plant life history. Because there is strong negative frequency dependence with regard to rare mating types in self-incompatible systems, new *S* alleles will be strongly self-propagating and thus easy to incorporate into recovery programs.

## Summary

Overall, our results highlight conditions under which mate limitation owing to self-incompatibility may be a significant constraint, not only on individual fecundity, but also for population persistence. However, this is likely to be the case for only a small parameter range of life histories (low *b*_o_ and high *d*) and under conditions where *S* allele diversity is relatively low. At extremes, when population growth rates are either robust or demographically very limited, genetic factors probably have only a small role to play in determining population viability. Our results also show that there can be direct links between genetic change and demographic performance in situations where self-incompatibility and *S* allele richness are important. Under these conditions, genetic remediation can be an effective management approach.

A crucial unexplored problem in conservation biology is to develop a predictive framework for identifying situations where genetic intervention is likely to be an effective strategy. Such situations may be hard to detect – for example, our results indicate that many measures of population “health” (e.g., *F*_IS_, variance in fitness) may show little response to changes in population size over a large range of values, but will drop off sharply when crucial thresholds are reached. Effective management depends on the ability to identify populations that superficially appear to be stable, but may decline rapidly in the future, given current trends (Thrall et al. 1997).

The model described here incorporates some of the demographic and genetic complexities of self-incompatibility in a spatially realistic setting. It is encouraging that comparison of model outcomes with data from long-term empirical studies of natural systems shows general qualitative and quantitative agreement. However, a number of issues warrant further study. First, local spatial genetic structure (and its dependence on the extent of pollen flow and seed dispersal) will be an important determinant of population dynamics and viability (Wells and Young 2002). Second, our model assumes simple linear dominance relationships among *S* alleles, but in nature, these interactions are often considerably more complex, potentially leading to quite different impacts of *S* allele richness on mate availability. Third, most empirical studies of natural systems indicate at least some potential for dissolution of SI systems (Busch and Schoen 2008), which raises the general issue (especially in the gametophytic case) of trade-offs between mate limitation and inbreeding depression which our analyses do not consider. Finally, our results indicate that the occasional addition of *S* alleles could be critical, thus pointing toward the importance of metapopulation processes and the potential for “genetic rescue” through interpopulation movement of seeds and pollen (Thrall et al. 2000). Such genetic rescue effects have been demonstrated for inbreeding (Richards 2000) and more recently suggested for *S* alleles (Wagenius et al. 2007).
